# Synergistic effects on itaconic acid production in engineered *Aspergillus niger* expressing the two distinct biosynthesis clusters from *Aspergillus terreus* and *Ustilago maydis*

**DOI:** 10.1186/s12934-022-01881-7

**Published:** 2022-08-11

**Authors:** Yaqi Wang, Yufei Guo, Wei Cao, Hao Liu

**Affiliations:** 1 MOE Key Laboratory of Industrial Fermentation Microbiology, College of Biotechnology, Tianjin University of Science & Technology, Tianjin, 300457 People’s Republic of China; 2 Tianjin Engineering Research Center of Microbial Metabolism and Fermentation Process Control, Tianjin University of Science & Technology, Tianjin, 300457 People’s Republic of China; 3 National Technology Innovation Center of Synthetic Biology, Tianjin, 300308 People’s Republic of China

**Keywords:** Itaconic acid production, *Aspergillus niger*, Metabolic engineering, Shake flask fermentation

## Abstract

**Background:**

Itaconic acid (IA) is a versatile platform chemical widely used for the synthesis of various polymers and current methods for IA production based on *Aspergillus terreus* fermentation are limited in terms of process efficiency and productivity. To construct more efficient IA production strains, *A. niger* was used as a chassis for engineering IA production by assembling the key components of IA biosynthesis pathways from both *A. terreus* and *Ustilago maydis*.

**Results:**

Recombinant *A. niger* S1596 overexpressing the *A. terreus* IA biosynthesis genes *cadA*, *mttA*, *mfsA* produced IA of 4.32 g/L, while *A. niger* S2120 overexpressing the *U. maydis* IA gene cluster *adi1*, *tad1*, *mtt1*, *itp1* achieved IA of 3.02 g/L. Integration of the two IA production pathways led to the construction of *A. niger* S2083 with IA titers of 5.58 g/L. Increasing *cadA* copy number in strain S2083 created strain S2209 with titers of 7.99 g/L and deleting *ictA* to block IA degradation in S2209 created strain S2288 with IA titers of 8.70 g/L. Overexpressing *acoA* to enhance the supply of IA precursor in strain S2288 generated strain S2444 with IA titers of 9.08 g/L in shake flask.

**Conclusion:**

Recombinant *A. niger* overexpressing the *U. maydis* IA biosynthesis pathway was capable of IA accumulation. Combined expression of the two IA biosynthesis pathways from *A. terreus* and *U. maydis* in *A. niger* resulted in much higher IA titers*.* Furthermore, increasing *cadA* copy number, deleting *ictA* to block IA degradation and overexpressing *acoA* to enhance IA precursor supply all showed beneficial effects on IA accumulation.

**Supplementary Information:**

The online version contains supplementary material available at 10.1186/s12934-022-01881-7.

## Background

Itaconic acid (IA), a C5-dicarboxylic acid containing a double bond, is a versatile platform chemical with a wide range of industrial applications such as production of superabsorbents, polyester resins, synthetic latex, surfactants [1] and IA is listed by DOE of U.S. as one of bio-based top 12 building block chemicals [2].

**Fig. 1:**
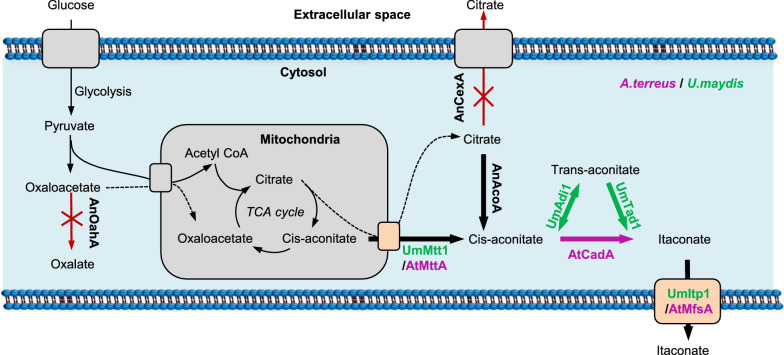
The constructed IA biosynthesis pathway in *A. niger*. Green represents relevant enzymes from *U. maydis*. Purple indicates specific enzymes from *A. terreus*. The “X” indicates gene deletion. For *A. niger* proteins, AnCexA: citrate exporter, AnOahA: oxaloacetate acetylhydrolase, AnAcoA: aconitase in cytosol; for *A.terreus* proteins, AtMttA: mitochondrial tricarboxylate transporter, AtCadA: *cis*-aconitate decarboxylase, AtMfsA: itaconic acid exporter; for *U. maydis*, UmAdi1: aconitate-Δ-isomerase, UmTad1: *trans*-aconitate decarboxylase, UmMtt1: mitochondrial tricarboxylate transporter, UmItp1: itaconic acid exporter

Although IA can be synthesized by conventional chemical means, commercial development is hampered due to the involvement of cumbersome and unsustainable process [3]. Diverse microorganisms are capable of producing IA, including *Aspergillus terreus* [4], *A. flavus* [5], *Ustilago maydis* [6], *Candida* sp [7] and *Pseudozyma antarctica* [8] etc. (Table 1). Strains of *A. terreus* and *U. maydis* have been extensively investigated for IA production. The metabolic pathways of IA biosynthesis for the two fungal species were distinct (Fig. 1) [3]. *A. terreus* utilizes *cis*-aconitate decarboxylase encoded by *cadA* to convert *cis*-aconitate to IA, while *U. maydis* uses two-step reactions involving aconitate-Δ-isomerase encoded by *adi1* and *trans*-aconitate decarboxylase encoded by *tad1* to form IA [6, 9]. Mitochondrial carrier proteins MttA and Mtt1 and transporter proteins MfsA and Itp1 are essential components of IA transport and thus the accumulation of IA in fermentation broth. MttA and MfsA genes are clustered in *A. terreus*, while Mtt1 and Itp1 genes are clustered in *U. maydis* [3]. MttA and Mtt1 mediate *cis*-aconitate transport from mitochondria to the cytoplasm, while MfsA and Itp1 are responsible for extracellular secretion of IA [3].

**Table 1:** Itaconic acid production in different microorganisms

Microorganism	Substrate	Production (g/L)	Yield (g/g)	Productivity (g/L/h)	pH	System	References
*Aspergillus terreus* NRRL 1962	Glucose	43.5	_	0.26	3.1	SF	[18]
	Xylose	31.6	_	0.19	3.1	SF	
	Arabinose	16.7	_	0.10	3.1	SF	
*Aspergillus terreus*	Glucose	86.2	0.62	0.51	3.1	BF	[19]
*Aspergillus terreus* DSM 23081	Glucose	92.4	0.53	_	3.1	SF	[20]
*Aspergillus terreus*	Glucose	160	0.46	0.99	3.4	BF	[4]
*Aspergillus terreus*	Glucose	146	_	_	3.0	BF	[21]
*Ustilago vetiveriae* TZI	Glycerol	34.7	0.18	0.09	6.5	SF	[22]
*Ustilago maydis* MB 215	Glucose	220	0.45	0.73	6.2	BF	[12]
*Escherichia coli*	Glucose	4.34	_	0.041	6.8	BF	[23]
*Escherichia coli*	Glucose	2.27	0.77	_	7.0	SF	[24]
	Glucose	32	0.68	0.45	7.0	BF	
*Saccharomyces cerevisiae*	Glucose	0.168	_	_	_	SF	[25]
*Saccharomyces cerevisiae*	Glucose	1.3	_	_	_	BF	[26]
*Yarrowia lipolytica*	Glucose	4.6	0.058	0.045	3.5 ~ 5.0	BF	[27]
*Carynebacterium glutamicum*	Glucose	7.8	0.4	0.0021	_	SF	[28]
*Aspergillus niger* ATCC 1015	Glucose	1.4	_	_	_	SF	[29]
*Aspergillus niger*	Sorbitol & xylose	7.1	_	0.091	_	BF	[16]
*Aspergillus niger* AB 1.13	Glucose	1.5	_	_	3.5	BF	[15]
*Aspergillus niger* H915-1	Glucose	4.92	_	_	3.1	SF	[30]
*Aspergillus niger*	Glucose	26.2	_	0.35	3.5	BF	[17]
	Glucose	2	_	_	_	SF	

*SF *shake flask, *BF* biofermenter

Though various microbial strains have been explored for fermentation production of IA [3], *A. terreus* is still the main source of strains for commercial IA production by fermentation. It was reported that a titer of 80 g/L IA was achieved by a genetically engineered industrial *A. terreus* with a yield of 0.6 g/g and productivity of 1.1 g/L/h [10]. Glucose is the most commonly used carbon source for IA fermentation by *A. terreus*, however, the low yield leads to high cost of IA biofabrication [11]. Additionally, much higher titers of IA fermentation process has been documented for engineered *U. maydis* strains with IA titers of 220 g/L and productivity of 0.73 g/L/h [12], but the low yield of 0.45 g/g prevented the use of *U. maydis* for IA commercial production [5]. *A. niger* has numerous desirable traits to be used as a microbial chassis cell for efficient IA production, including a high conversion efficiency from sugar to organic acid, a clear genetic background, low pH tolerance and capable of utilizing cheap and renewable carbon sources [13]. Previously, it was shown that recombinant *A. niger* overexpressing *A. terreus cadA* encoding the *cis*-aconitate decarboxylase was capable of IA production [14]. Moreover, combined overexpression of *cadA/ mttA* or *cadA/ mfsA* in *A. niger* led to enhanced IA production with titers of 1.5 g/L [15]. Overexpressing *cadA*, *mttA* and *mfsA* in an oxaloacetate hydrolase and glucose oxidase deletion mutant *A. niger* NW186 further boosted IA yields, reaching up to 7.1 g/L [16]. Overexpression of cytosolic citrate synthase (*citB*) in *A. niger* carrying the IA-producing gene cluster of *A. terreus* achieved IA titers of 2 g/L and 26.2 g/L in shake flask and controlled batch cultivation, respectively [17]. Although IA production in *A. niger* expressing the IA biosynthesis pathway genes from *A. terreus* were examined by several studies, the effects of introducing genes from *U. maydis* IA biosynthesis pathway into *A. niger* on IA production has yet to be reported. It is worth investigating the potential beneficial effects that integrates the positive genes from IA biosynthesis pathways of *A. terreus* and *U. maydis* into the genome of *A. niger* (Fig. 1).

In this study, an engineered *A. niger* strain S1075 deficient in citrate extracellular secretion (*cexA* deletion) and oxalate production (*oahA* deletion) was used as starting host strain for assembling the IA biosynthesis pathways. First, *cadA*, *mttA* and *mfsA* from *A. terreus* were sequentially introduced into S1075 to obtain IA-producing recombinant strain S1596, and *adi1*, *tad1*, *mtt1* and *itp1* from *U. maydis* were sequentially introduced into *A. niger* S1075 to obtain IA-producing strain S2120. Second, *adi1*, *tad1* and *itp1* were integrated sequentially into strain S1596 to obtain IA-producing strain S2083. Finally, an efficient IA-producing *A. niger* cell factory S2444 was constructed by increasing *cadA* gene copy number, overexpressing *acoA* to enhance supply of IA precursor and deleting *ictA* to block IA degradation in strain S2083.

## Results and discussion

### Expression of the A. terreus IA biosynthesis cluster in A. niger S1075

**Fig. 2:**
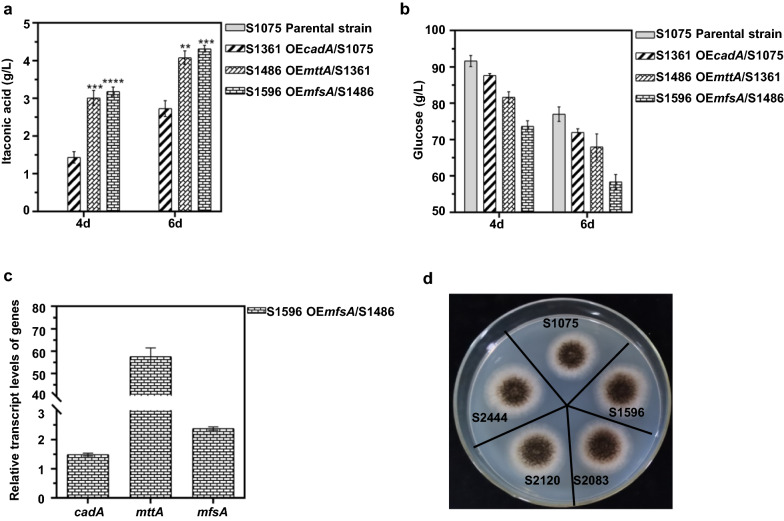
Construction of *A. niger* cell factory overexpressing specific genes of IA synthesis cluster from *A. terreus*. **a** The titer of IA generated by the indicated strains in shake flasks for 4 days and 6 days. **b** The residual glucose obtained for the indicated strains in shake flasks for 4 days and 6 days. **c** The determination of expression levels of genes involved in IA production by qRT-PCR in S1596. **d** Growth of parent strain and mutant strains on PDA plate at 28 °C. Mean and standard deviation values were calculated from 3 independent Erlenmeyer flask samples. The “*” represents statistically significant difference by one-tailed Student’s test. OE represents overexpression

Naturally occurring strains of *A. niger* are incapable of producing IA due to the absence of *cis*-aconitate decarboxylase [31]. The fact that engineered *A. niger* strains expressing a *cis*-aconitate decarboxylase from *A. terreus* led to IA accumulation showed the potential of *A. niger* as a microbial chassis for IA production [14, 29, 31, 32]. In this study, an oxalate-non-producing strain *A. niger* S422 derived from *A. niger* ATCC 1015 [33] was used as the parent strain to delete the citrate exporter-coding gene *cexA* to prevent citrate accumulation during IA fermentation. The resultant citrate-non-producing strain was named S1075. In this study, a *cis*-aconitate decarboxylase gene *cadA* from *A. terreus* was first introduced into *A. niger* S1075. Thirty-five and ten transformants overexpressing *cadA* were selected for IA fermentation analysis in the first and second round of screening, respectively. Similar levels of IA accumulation were found in all transformants (Additional file 1: Fig. S2a). The transformant with highest IA titers (1.44 g/L and 2.73 g/L at 4-day and 6-day, respectively) was named as S1361 (Fig. 2a). Our results demonstrated that introduction of *cadA* to *A. niger* confers its ability to biosynthesize IA. Similar results were also observed in other engineered *A. niger* strains [14, 16].

To increase the supply of *cis*-aconitate for decarboxylation reaction in the cytosol, *mttA* encoding a mitochondrial tricarboxylate transporter responsible for aconitate transport across mitochondrial membrane was overexpressed in S1361. We next introduced *mttA* and tested the resulting strain S1486 as described above (Additional file 1: Fig. S3). As shown in Fig. 2a, overexpression of *mttA* further elevated IA production to 3.01 g/L and 4.08 g/L at 4-day and 6-day, respectively.These results demonstrated that efficient aconitate transport into cytosol benefited IA production, which is consistent with the previous observations that transport of *cis*-aconitate out of mitochondria is one of the limiting steps in IA production [15].

Furthermore, export of IA out of cytosol is previously shown to be a pivotal step for IA accumulation [15], which is mediated by the major facilitator superfamily protein MfsA as IA exporter in *A. terreus*. For IA fermentation analysis, transformants overexpressing *mfsA* were selected as described above, and similar levels of IA accumulation were found in all transformants (Additional file 1: Fig. S4a, b). The transformant with highest IA titer (3.18 g/L and 4.32 g/L at 4-day and 6-day, respectively) was named as S1596 (Fig. 2a). The above results indicated that the gene cluster of IA biosynthesis from *A. terreus* has a significant positive effect on the accumulation of IA in *A. niger* [17]. Additionally, as important precursor substances for IA biosynthesis, intracellular *cis*-aconitate concentration was also determined. As shown in Additional file 1: Table S1, intracellular *cis*-aconitate slightly increased in S1596 compared with that in *A. niger* S1075, suggesting that the metabolic flux to IA was enhanced by introducing the gene cluster of IA biosynthesis from *A. terreus*. Additionally, a small amount of *trans*-aconitate was formed in *A. niger* S1596 (Additional file 1: Table S1), which may be due to the lower thermal stability of the *cis*-aconitate than *trans*-aconitate, prompting the isomerization of *cis*-aconitate into *trans*-aconitate [34]. The results showed that overexpression of *cadA*, *mttA* and *mfsA* leads to the intracellular conversion of *cis*-aconitate into IA. As the levels of IA accumulation continued to increase, the rates of glucose consumption also increased (Fig. 2b). The results demonstrated that the introduction of *cadA*, *mttA* and *mfsA* genes successfully engineered the metabolic flux to IA, which indicated that the IA biosynthesis pathway derived from *A. terreus* worked and exhibited great potential for IA production in *A. niger* chassis. The overexpression of *cadA*, *mttA* and *mfsA* in *A. niger* S1596 was verified by qRT-PCR (Fig. 2c).

### Expression of the U. maydis IA biosynthesis cluster in A. niger S1075

**Fig. 3:**
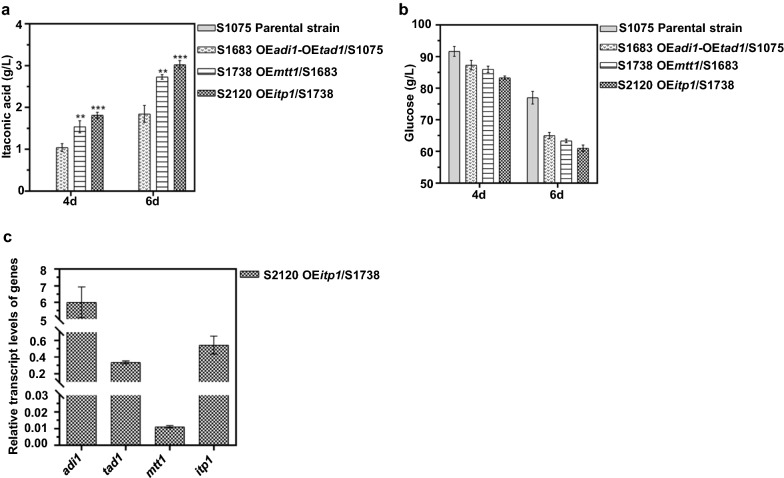
Construction of *A. niger* cell factory expressing relevant genes from *U. maydis* to produce IA. **a** The titer of IA generated by the indicated strains in shake flasks for 4 days and 6 days. **b** The residual glucose obtained for the indicated strains in shake flasks for 4 days and 6 days. **c** The determination of expression levels of genes involved in IA production by qRT-PCR in S2120. Mean and standard deviation values were calculated from 3 independent Erlenmeyer flask samples. The “*” represents statistically significant difference by one-tailed Student’s test. OE represents overexpression

*U. maydis* is a natural organism to producing dicarboxylic acid using an alternative pathway via *trans*-aconitate [35]. The aconitate-Δ-isomerase (Adi1) and *trans*-aconitate decarboxylase (Tad1) from *U. maydis* are central for IA biosynthesis, and Tad1 is related to 3-carboxy-cis, *cis*-muconate lactonizing enzyme and Adi1 is a member of the PrpF family [6]. In *S. cerevisiae*, IA could be synthesized only when the two genes *adi1* and *tad1* were co-expressed [6]. To evaluate whether introducing the *U. maydis* gene cluster into *A. niger* is capable of producing IA, the aconitate-Δ-isomerase gene *adi1* and *trans*-aconitate decarboxylase *tad1* were first co-overexpressed in *A. niger* S1075. According to the method described above, transformants co-overexpressing *adi1* and *tad1* were selected (Additional file 1: Fig. S5a, b). *A. niger* S1683 co-overexpressing *adi1* and *tad1* was shown to be the best IA-producing strain with IA titers of 1.04 g/L and 1.85 g/L IA at 4-day and 6-day, respectively (Fig. 3a), which further illustrated that both *adi1* and *tad1* played important roles in IA production in *A. niger*.

It is well known that isomerization of *cis*-aconitate and decarboxylation of *trans*-aconitate occur in the cytosol, and the putative mitochondrial transporter Mtt1 is the rate-limiting step in the transport of *cis*-aconitate from mitochondria to cytosol [6]. Thus, we further introduced *mtt1* and selected the recombinant strain S1738 with the highest IA titer as described above (Additional file 1: Fig. S6a, b). As shown in Fig. 3a, *mtt1* overexpression promotes IA formation in *A. niger* S1738 with titers of 1.54 g/L and 2.74 g/L at 4-day and 6-day, respectively, indicating an enhanced metabolic flux of *cis*-aconitate to cytosol and thus an acceleration of bioconversion of *cis*-aconitate to *trans*-aconitate.

Considering that a major facilitator superfamily transporter Itp1 was previously shown to be responsible for the IA transport [6], thus we overexpressed *itp1* in strain S1738. Transformants overexpressing *itp1* were selected as described above, and transformant with highest IA titers named as S2120 (OE*adi1*, OE*tad1*, OE*mtt1*, OE*itp1*) (Additional file 1: Fig. S7a, b). As shown Fig. 3a, titers of IA in S2120 fermentation broth were 1.82 g/L (0.07 mol/mol) and 3.02 g/L (0.08 mol/mol) at 4-day and 6-day, respectively, which was almost twice that of strain S1683. The results suggested that introduction of *itp1* further strengthened the efficiency of IA transport, indicating that IA export was an indispensable factor for the elevation of IA accumulation. Furthermore, compared with *A. niger* S1075, comparable levels of *cis*-aconitate and more *trans*-aconitate were detected in *A. niger* S2120 (Additional file 1: Table S1), suggesting that the gene cluster of IA biosynthesis from *U. maydis* successfully enhanced the metabolic flux of *cis*-aconitate to IA in *A. niger* through *cis*/*trans*-aconitate isomerization. The levels of residual sugar also decreased as IA production increased (Fig. 3b). Overexpression of *itp1* may drive the metabolic flux toward IA biosynthesis, making more carbon sources available for IA production. Monitoring of the transcript levels also demonstrated that *adi1*, *tad1*, *mtt1* and *itp1* were overexpressed and have played their expected roles in promoting IA production in strain S2120 (Fig. 3c).

### Expression of the U. maydis IA biosynthesis cluster in strain S1596

**Fig. 4:**
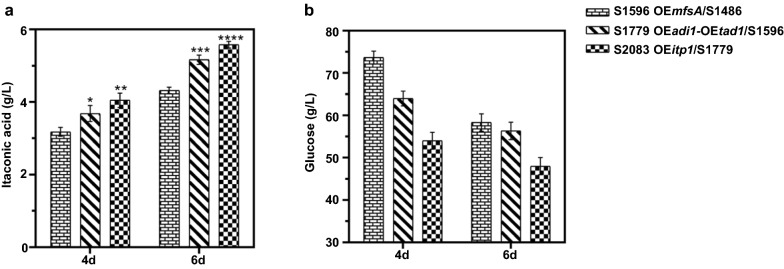
IA production in *A. niger* S1596 overexpressing genes of the IA sythesis cluster from *U. maydis*. **a** The titer of IA produced by the indicated strains in shake flasks for 4 days and 6 days. **b** The residual glucose obtained for the indicated strains in shake flasks for 4 days and 6 days. Mean and standard deviation values were calculated from 3 independent Erlenmeyer flask samples. The “*” represents statistically significant difference by one-tailed Student’s test. OE represents overexpression

In order to further improve IA production in *A. niger*, we investigated the potential beneficial effects for IA synthesis by integrating the two IA biosynthesis routes from *A. terreus* and *U. maydis*. Given that IA titers of S1596 (OE*cadA*, OE*mttA*, OE*mfsA*) were significantly higher than that of S2120 (OE*adi1*, OE*tad1*, OE*mtt1*, OE*itp1*) (Figs. 2a, 3a), *adi1* and *tad1* were co-introduced into S1596. For IA fermentation analysis, transformants co-overexpressing *adi1* and *tad1* were selected and tested as described above (Additional file 1: Fig. S8a, b). The transformant with the highest IA titer was selected for further engineering and named as S1779 (OE*cadA*, OE*mttA*, OE*mfsA,* OE*adi1*, OE*tad1*) with titers of 3.68 g/L (0.09 mol/mol) and 5.17 g/L (0.11 mol/mol), respectively (Fig. 4a). CAD was a key enzyme for IA production in *A. terreus* [14], while ADI and TAD played important roles in *cis*-aconitate isomerization to *trans*-aconitate as well as IA biosynthesis in *U. maydis* [6]. The above results showed that co-overexpression of *adi1* and *tad1* in *A. niger* cells containing the IA biosynthesis pathway from *A. terreus* further enhanced the metabolic flux to IA, which exhibited synergistic effects on IA production.

Previous studies demonstrated transporter Itp1 is a rate-limiting step for IA production in *U. maydis* [6]. Therefore, *itp1* was inserted into S1779 in the hope of alleviating this metabolic flux bottleneck. We next introduced *itp1* in S1779 and tested the resulting strains S2083 as described above (Additional file 1: Fig. S9a, b). As expected, IA titers in *A. niger* S2083 increased to 3.97 g/L (0.12 mol/mol) and 5.58 g/L (0.15 mol/mol) at 4-day and 6-day, respectively (Fig. 4a). As shown in Fig. 4b, glucose levels in fermentation broth also decreased with increased IA production, indicating that more carbon sources were directed to the IA biosynthesis pathway in *A. niger* S2083.

### Increasing the copy number of cadA in strain S2083

**Fig. 5:**
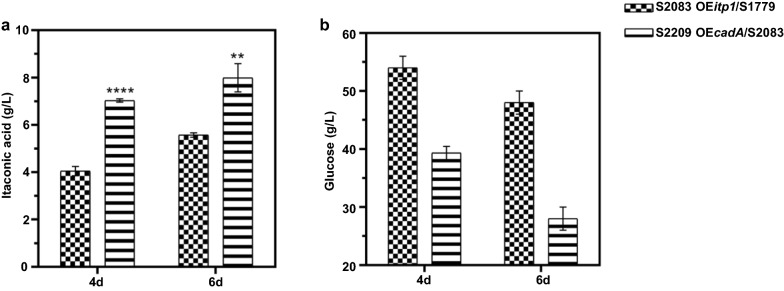
IA production in strain S2083 with increased *cadA* gene copy number. **a** The titer of IA generated by the indicated strains in shake flasks for 4 days and 6 days. **b** The residual glucose obtained for the indicated strains in shake flasks for 4 days and 6 days. Mean and standard deviation values were calculated from 3 independent Erlenmeyer flask samples. The “*” represents statistically significant difference by one-tailed Student’s test. OE represents overexpression

Increased copy number of *cadA* was shown to be beneficial for IA accumulation [36]. The titers of IA in all transformants with introduction of multiple copies of *cadA* exhibited higher titers of IA than *A. niger* S2083 (Additional file 1: Fig. S10a). After a second round of screening, the transformant with highest IA titer was selected and named as S2209 [OE*cadA* (multiple copies), OE*mttA*, OE*mfsA,* OE*adi1*, OE*tad1*, OE*itp1*] (Additional file 1: Fig. S10b). As shown Fig. 5a, titers of IA in *A.niger* S2209 fermentation broth were 7.04 g/L and 7.99 g/L at 4-day and 6-day, respectively. It has been reported that the expression level of CAD was proportional to the IA production [16, 23]. Indeed, a similar phenomenon was observed in this study, suggesting that the conversion *cis*-aconitate to IA was a rate-limiting step for IA biosynthesis in the heterogenously hybrid cell factory. Additionally, the concentration of glucose also decreased with increased IA production (Fig. 5b). In conclusion, the incorporation of multiple copies of *cadA* into *A. niger* promoted the decarboxylation reaction of aconitic acid, thus enhancing its biosynthesis of IA.

### Reduced byproduct itaconyl-CoA accumulation in strain S2209

**Fig. 6:**
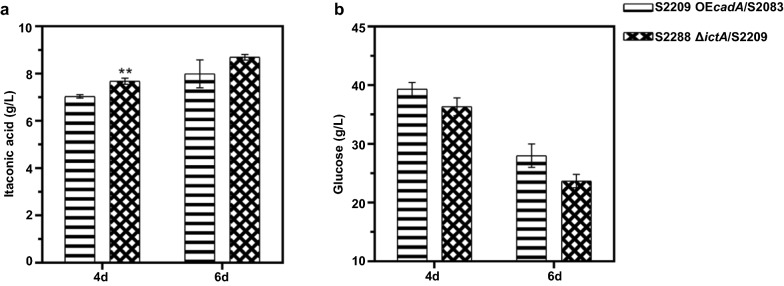
IA production in strain S2209 with *ictA* deletion. **a** The titer of IA generated by the indicated strains in shake flasks for 4 days and 6 days. **b** The residual glucose obtained for the indicated strains in shake flasks for 4 days and 6 days. Mean and standard deviation values were calculated from 3 independent Erlenmeyer flask samples. The “*” represents statistically significant difference by one-tailed Student’s test. OE represents overexpression. Δ represents deletion

Itaconyl-CoA transferase (IctA) was shown to convert IA to itaconyl-CoA in *A. niger*, the step of IA degradation pathway [37]. To prevent the IA degradation, *ictA* encoding itaconyl-CoA transferase was deleted in S2209 to obtain recombinant strain S2288 [OE*cadA* (multiple copies), OE*mttA*, OE*mfsA*, OE*adi1*, OE*tad1*, OE*itp1*, Δ*ictA*]. As shown in Fig. 6a, deletion of *ictA* resulted in IA accumulation with titers of 7.68 g/L and 8.70 g/L at 4-day and 6-day, respectively, indicating that *ictA* deletion is beneficial for IA accumulation. Additionally, Fig. 6b showed that the levels of residual sugar for strain S2288 was lower than that of S2209 in shake flask fermentation (Fig. 6b). These results indicate that blocking itaconyl-CoA formation likely directed more carbon for IA biosynthesis.

### Construction of an efficient IA-producing recombinant strain S2444

**Fig. 7:**
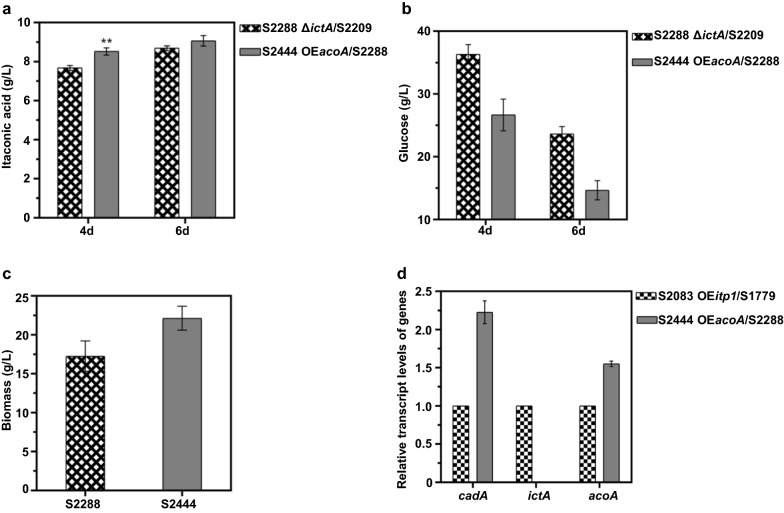
IA production in strain S2288 overexpressing *acoA* in cytosol. **a** The titer of IA generated by the indicated strains in shake flasks for 4 days and 6 days. **b** The residual glucose obtained for the indicated strains in shake flasks for 4 days and 6 days. **c** The biomass measured by collecting mycelium of 6-days cultured in IA fermentation medium. **d** The determination of expression levels of genes involved in IA production by qRT-PCR in the control strain S2083 and strain S2444. Mean and standard deviation values were calculated from 3 independent Erlenmeyer flask samples. The “*” represents statistically significant difference by one-tailed Student’s test. OE represents overexpression. Δ represents deletion

*Cis*-aconitic acid as a precursor of IA is formed by the dehydration of citric acid under the action of aconitase encoded by *acoA* [2]. Aconitase is mainly found in mitochondria, while *cis*-aconitic acid decarboxylase, aconitate-Δ-isomerase and *trans*-aconitate decarboxylase are located in the cytosol [35]. The positive effect of overexpression of the aconitase was also shown in *A. niger* [29] and *E. coli* [38]. Thus, *acoA* was overexpressed in the cytosol of S2288 to obtain more efficient IA-producing strain. As mentioned above, we next introduced the gene *acoA* into S2288 to create recombinant strain S2444 (Additional file 1: Fig. S11a, b). The overexpression of *acoA* enabled further accumulation of IA in *A. niger* S2444 with titers of 8.53 g/L and 9.08 g/L, respectively (Fig. 7a). *A. terreus* is a natural producer of IA. However, the relative high price of IA produced by *A. terreus* was partly due to its high purity requirements of industrial cultivation medium and sophisticated control of fermentation processes [39]. Recently, *U. maydis* was reported to be capable of IA accumulation with long fermentation period and low productivity [12]. By contrast, *A. niger* is an outstanding workhorse of organic acid production with low requirements for fermentation nutrition and excellent acid resistance. Additionally, *A. niger* is a robust cell factory for the accumulation of citric acid, an important precursor of IA biosynthesis, with high productivity and low cost.

*acoA* overexpression strain S2444 exhibited similar growth to S1075, S1596 and S2120 on PDA medium (Fig. 2d). Moreover, the glucose in S2444 fermentation broth was almost depleted (Fig. 7b), indicating that more carbon was converted to IA. These results were similar to previous studies, in which malic acid production was improved by reinforcing glycolytic pathway and intensifying glucose transport [40]. Compared with the starting strain S2288, the biomass of strain S2444 increased by 28.20% in shake flask fermentation (Fig. 7c). Additionally, we analyzed the transcript levels for 3 genes involved in IA production. Compared with strain S2083 overexpressing *cadA* and *acoA*, the expression levels of these two genes were increased by 2.23 and 1.55 folds in S2444, respectively, and transcripts for *ictA* were not detected (Fig. 7d).

The identification and functional analysis of key genes for IA biosynthesis and the metabolic engineering in the hosts, including *A. terreus*, *U. maydis* etc., provided valuable insights for guiding the construction of an *A. niger* cell factory for efficient IA production. Theoretically, the extrapolations are often tentative, organism specific and purely qualitative. Therefore, quantitative metabolic flux predictions based on the genetic modifications of the key components of IA biosynthesis and secretion pathways would further guide the rational design of designer strains of *A. niger* for more efficient IA production. Although directing more metabolic flux to citric acid and/or aconitic acid has been shown to enhance IA accumulation in recombinant *A. niger*, a quantitative model relating metabolic flux to IA titers is still lacking. Construction of a quantitative metabolic flux prediction model for IA titers will entail the measurement of the concentrations of key metabolites including citrate, *cis*-aconitate in both cytoplasm and mitochondria, as well as quantitative measurements of the expression levels of key relevant genes in the IA biosynthesis and secretion pathways of in engineered *A. niger* strains, thus accelerating the design and engineering of more efficient IA production strains using the *A. niger* chassis. Additionally, MES was used to maintain the pH in IA production in flask cultures in this study. Considering the cost of using high concentrations of MES, recently we further evaluated the potential of the recombinant strains to synthesize IA in fermentation tanks in which the pH was adjusted in real time by feeding NaOH. Our primary data indicated that the recombinant strains, such as S2444, produced titers of IA using NaOH as pH neutralizer comparable to that of using MES (data not reported). Thus, NaOH would provide a practical means to maintain a desirable pH for IA fermentation with our engineered strains. In fact, NaOH is widely used as a pH neutralizer in industry due to its efficiency and low cost.

## Conclusion

*A. niger* is a powerful and versatile cell factory for industrial production of organic acids such as citric acid and malic acid. Engineering efficient *A*. *niger* cell factories for development of fermentation for commercial IA production represents an important research direction. Given that natural *A. niger* strains do not produce IA, engineering *A. niger* for efficient IA production depends on the construction of artificial IA biosynthesis route by recruiting and tuning of key components of IA synthetic pathways from other microorganisms. In this study, genes from IA biosynthesis clusters of *A. terreus* and *U. maydis* were overexpressed individually or in various combinations in *A. niger.* Our results showed that overexpressing *adi1*, *tad1*, *mtt1* and *itp1* from the *U. maydis* IA biosynthesis gene cluster enabled the production in *A. niger*, and combined expression of the two distinct IA biosynthesis gene clusters in strain S2083 (OE*cadA*, OE*mttA*, OE*mfsA*, OE*adi1*, OE*tad1*, OE*itp1*) demonstrated higher IA production than expression of either of the two biosynthesis routes. Furthermore, increasing the *cadA* copy number, blocking the IA degradation by *ictA* deletion and overexpressing *acoA* in *A. niger* all contributed to enhanced IA production in an additive manner, as shown in the recombinant strain S2444 [OE*cadA* (multiple copies), OE*mttA*, OE*mfsA*, OE*adi1*, OE*tad1*, OE*itp1*, Δ*ictA*, *OEacoA*] achieving IA titers of 9.08 g/L.

## Materials and methods

### Strains and culture conditions

*A. niger* S422 derived from *A. niger* ATCC 1015 [33] was used as the parent strain to delete the citrate exporter-coding gene *cexA* to prevent citrate accumulation during IA fermentation. The resultant citrate-non-producing strain was named S1075. *A. niger* strains were grown at 28 °C on potato dextrose agar (PDA) supplemented with or without 250 μg/mL hygromycin B as previously described [40]. Selection for *A. niger* transformants was performed in the medium A composed of 30 g/L malt extract, 5 g/L tryptone, 218 g/L sorbitol, 20 g/L agar [41]. The recombination of *lox*P-*hph*-*lox*P loci was achieved in the PDA plates supplemented with 10 μg/mL doxycycline (DOX) as previously reported with slight modifications. *Escherichia coli* JM109 used for gene cloning was cultured at 37 °C in Luria Bertani (LB) medium [42]. *A. niger* mutants obtained in this study are listed in Table 2.

**Table 2:** Strains and plasmids used in this study

Strain or plasmid	Genotype/description	Source
*Strains*		
S422	Tet-On::*cre*, Δ*oahA*	[33]
S1075	Tet-On::*cre*, Δ*oahA*, Δ*cexA*	This study
S1361	Tet-On::*cre*, Δ*oahA*, Δ*cexA*, OE*cadA*	This study
S1486	Tet-On::*cre*, Δ*oahA*, Δ*cexA*, OE*cadA*, OE*mttA*	This study
S1596	Tet-On::*cre*, Δ*oahA*, Δ*cexA*, OE*cadA*, OE*mttA*, OE*mfsA*	This study
S1683	Tet-On::*cre*, Δ*oahA*, Δ*cexA*, OE*adi1*, OE*tad1*	This study
S1738	Tet-On::*cre*, Δ*oahA*, Δ*cexA*, OE*adi1*, OE*tad1*, OE*mtt1*	This study
S1779	Tet-On::*cre*, Δ*oahA*, Δ*cexA*, OE*cadA*, OE*mttA*, OE*mfsA*, OE*adi1*, OE*tad1*	This study
S2083	Tet-On::*cre*, Δ*oahA*, Δ*cexA*, OE*cadA*, OE*mttA*, OE*mfsA*, OE*adi1*, OE*tad1*, OE*itp1*	This study
S2120	Tet-On::*cre*, Δ*oahA*, Δ*cexA*, OE*adi1*, OE*tad1*, OE*mtt1*, OE*itp1*	This study
S2209	Tet-On::*cre*, Δ*oahA*, Δ*cexA*, OE*cadA* (multiple copies), OE*mttA*, OE*mfsA*, OE*adi1*, OE*tad1*, OE*itp1*	This study
S2288	Tet-On::*cre*, Δ*oahA*, Δ*cexA*, OE*cadA* (multiple copies), OE*mttA*, OE*mfsA*, OE*adi1*, OE*tad1*, OE*itp1*, Δ*ictA*	This study
S2444	Tet-On::*cre*, Δ*oahA*, Δ*cexA*, OE*cadA* (multiple copies), OE*mttA*, OE*mfsA*, OE*adi1*, OE*tad1*, OE*itp1*, Δ*ictA*, OE*acoA*	This study
*Plasmids*		
pLH257	P*glaA*, *cadA* gene, T*trpC*, *ble*^*r*^, *kan*^*r*^	This study
pLH454	*lox*P-*hph-lox*P, P*gpdA*, T*trpC*, *hyg*^*r*^, *kan*^*r*^	[40]
pLH509	*lox*P-*hph-lox*P, Pp*kiA*, T*trpC*, *hyg*^*r*^, *kan*^*r*^	[40]
pLH594	*lox*P-*hph-lox*P, P*gpdA*, T*trpC*, *hyg*^*r*^, *ppt*^*r*^, *kan*^*r*^	[40]
pLH780	*lox*P-*hph-lox*P, P*gpdA*, *acoA* gene, T*trpC*, *hyg*^*r*^, *kan*^*r*^	This study
pLH786	*lox*P-*hph-lox*P, P*gpdA*, *cadA* gene, T*trpC*, *hyg*^*r*^, *kan*^*r*^	This study
pLH850	*lox*P-*hph-lox*P, P*gpdA*, *mttA* gene, T*trpC*, *hyg*^*r*^, *kan*^*r*^	This study
pLH862	*lox*P-*hph-lox*P, P*gpdA*, m*fsA* gene, T*trpC*, *hyg*^*r*^, *kan*^*r*^	This study
pLH942	*lox*P-*hph-lox*P, P*gpdA*, *adi1* gene, P*pkiA*, *tad1* gene, T*trpC*, *hyg*^*r*^, *kan*^*r*^	This study
pLH991	*lox*P-*hph-lox*P, Pp*kiA*, *itp1* gene, T*trpC*, *hyg*^*r*^, *kan*^*r*^	This study
pLH1008	*lox*P-*hph-lox*P, 5’ and 3’ flanking sequences of *ictA*, ppt^r^, *kan*^*r*^	This study
pLH1023	*lox*P-*hph-lox*P, P*gpdA*, *mtt1* gene, T*trpC*, *hyg*^*r*^, *kan*^*r*^	This study

*hyg*^*r*^ represents hygromycin B resistance, *kan*^*r*^ indicates kanamycin resistance, *ppt*^*r*^ indicates phosphinothricin resistance, Δ represents knockout, *OE* represents overexpression

### Plasmid construction

To express *cadA* derived from *A. terreus* in *A. niger*, *cadA* was amplified by PCR with primers 3501/3502 using pLH257 as a template, which contains the *cadA* gene synthesized by GENEWIZ. The *Kpn* I-digested PCR product was purified and ligated to the downstream of glyceraldehyde-3-phosphate dehydrogenase promoter (P*gpdA*) of pLH454. The resultant recombinant plasmid was named pLH786. The same strategy was applied to construction of recombinant plasmids for expression of *mttA*, *mfsA*, *mtt1*, *itp1* and co-expression of *adi1* and *tad1*, respectively. The resultant recombinant plasmids were named pLH850, pLH862, pLHl023, pLH991 and pLH941, respectively. The expression of *mttA*, *mfsA*, *mtt1* and *adi1* was controlled by P*gpdA*, while the expression of *tad1* and *itp1* were under the control of promoter P*pkiA*, a pyruvate kinase promoter of *A. niger* [40]. The cytoplasmic expression of AcoA was constructed by removing its putative mitochondrial localization signal with a 24 amino acid N-terminal, and the resultant recombinant plasmid carrying the *acoA* expression cassette was named pLH780. The *ictA* gene deletion plasmid was constructed by the same method as previously described [40]. Briefly, the 5’ and 3’ flanking sequences of *ictA* were respectively amplified with primers 3522/3523 and primers 3524/3525 using *A. niger* genomic DNA as a temple. The relevant sequences were digested and integrated into the flanks of *lox*P-*hph*-*lox*P to obtain plasmid pLH1008. The same strategy was applied to the construction of recombinant plasmids for deletion of *cexA* and *oahA*, respectively. All primers used in this study are listed in Additional file 1: Table S2.

### Transformation of A. niger

Protoplast preparation was performed as previously described [43]. *A. niger* spores (1 × 10^8^) were inoculated into 250 mL of liquid fermentation medium (20 g/L glucose, 10 g/L yeast extract, 1 g/L MgSO_4_, 1 g/L KH_2_PO_4_) and cultivated for 16 ~ 18 h at 28 °C. The mycelia were harvested by centrifugation at 12,000 rpm for 15 min and washed with sterile water. Protoplast formation was achieved in 10 mL of MC buffer (7.35 g/L CaCl_2_, 3.90 g/L Mes, 52.19 g/L KCl, pH5.8) containing cell wall digesting enzymes including lysing enzymes from *Trichodema harzianum* (Sigma®, L1412), yatalase (Takara) and ß-glucuronidase from *Helix pomatia* (Sigma G7017). Incubation was performed at 37 °C with 120 rpm for 4 h. To the resulting protoplasts, 10 mL cold STC buffer (218.6 g/L sorbitol, 7.35 g/L CaCl_2_, 1.21 g/L Tris, pH7.5) was added and centrifuged at 2,300 rpm at 4 °C for 10 min, followed by resuspending twice with STC buffer and directly used for transformation of *A. niger*. Plasmid DNA (50 μg) was mixed with 200 μL of protoplast suspension and 660 μL PEG solution (600 g/L PEG6000, 147.02 g/L CaCl_2_), and incubated on ice for 20 min, followed by addition of 2 mL PEG solution and incubation for 10 min at room temperature. The protoplast mixture was spread on the medium A with 4 mL of STC buffer and 4 mL of the medium B composed of 30 g/L malt extract, 5 g/L tryptone, 218 g/L sorbitol, 10 g/L agar, and cultivated for 4 ~ 6 days at 28 °C. Fungal colonies appearing on PDA supplemented with 250 μg/mL hygromycin B were selected and further verified by PCR analysis using specific primers [40].

### Construction of target gene overexpression and deletion strains

Gene deletion and overexpression strains of *A. niger* were obtained by the polyethylene glycol (PEG)-mediated protoplast transformation system as described previously [43]. Briefly, plasmid DNA harboring *cadA* was integrated into *A. niger* S1075 genome through PEG-protoplast transformation. Transformants were selected on medium A supplemented with 250 μg/mL hygromycin B at 28 °C for 3 ~ 5 days and *cadA* integration event was determined by PCR analysis using primers 3501/641, and the generated OE*cadA* with *lox*P-*hph-lox*P cassette was named S1361. qRT-PCR was used to further determine gene overexpression or knockout in the mutants. As previously described, the hygromycin B sensitive strains were selected on PDA plates supplemented with 10 μg/mL DOX and verified via PCR analysis. The same strategy was used to construct *mttA*, *mfsA*, *adi1*, *tad1*, *mtt1*, *itp1* and *acoA* overexpression strains and *ictA* deletion strains. All strains in this study are listed in Table 2.

### Shake flask cultivation

For *A. niger* cultivation, 1 × 10^8^ spores were added to 50 mL IA fermentation medium in 250 mL baffled Erlenmeyer flask and cultivated at 35 °C on rotary shaker of 250 rpm for 6 days. The medium used IA fermentation was previously described Vogel’s medium with slight modification [32]. IA fermentation medium was composed of 100 g/L glucose, 2.5 g/L Na_3_-citrate × 2H_2_O, 5 g/L KH_2_PO_4_, 2 g/L NH_4_NO_3_, 0.2 g/L MgSO_4_ × 7H_2_O, 0.1 g/L CaCl_2_ × H_2_O, 0.5 mg/L citric acid × H_2_O, 0.5 mg/L ZnSO_4_ × 7H_2_O, 0.1 mg/L Fe(NH_4_)_2_(SO_4_)_2_ × 7H_2_O, 0.025 mg/L CuSO_4_ × 5H_2_O, 0.005 mg/L MnSO_4_ × H_2_O, 0.005 mg/L H_3_BO_3_, 0.005 g/L Na_2_MoO_4_ × 2H_2_O, and 0.005 mg/L biotin without MnSO_4_. Furthermore, IA fermentation medium contains 1 M morpholinoethanesulfonic acid (MES), and the starting pH of the medium was 6.5.

### Measurement of organic acid and glucose concentrations

The IA was detected using high-performance liquid chromatography (HPLC). The samples were centrifuged at 12,000 rpm. The supernatant was then diluted to 1:10 with ultrapure water and filtered through a 0.22 μm filter. The processed solution was measured by HPLC (Agilent 1260 VWD UV detector) applying Aminex HPX-87H column (Bio-Rad) thermostated at 65 °C [40]. This solution was monitored at wavelength 210 nm using a UV detector, with 5 mM H_2_SO_4_ as the mobile phase running at 0.6 mL/min based on the methods described previously [40]. The concentrations of each glucose were determined using SBA-40E biosensor analyzer (Biology Institute of Shandong Academy of Sciences, Jinan, China) [40].

### Biomass measurement

Biomass analysis was determined according to the literature with slight modification [44]. Briefly, 10 mL of cultures in shake flasks was harvested at the end of fermentation. Then, collected samples were washed with distilled water and filtered, and dried at 85 °C to a constant weight to obtain cell dry weight.

### RNA purification and transcription analysis

Spores (1 × 10^8^) of *A. niger* strains were added to 50 mL IA fermentation medium in 250 mL Erlenmeyer flasks at 35 °C, 250 rpm. Mycelia were collected after 6 days of cultivation and ground with liquid nitrogen. Extractions of total RNA were performed using E.A.N.A.™ Fungal RNA Kit (Omega Bio-tek, Inc.) and treated with OBI DNase I (Omega Bio-tek, Inc.) according to the manufacturer’s instructions. The cDNA was obtained by PrimeScript^TM^RT reagent Kit with gDNA Eraser Perfect Real Time (TaKaRa Biomedical Technology Co., Ltd). Quantitative real-time PCR (qRT-PCR) was performed on the Applied Biosystems StepOne TM Real-Time PCR System Thermal Cycling Block according to the manufacturer’s protocol. The calculated threshold cycle (Ct) values of the beta-actin gene was used as the corresponding endogenous reference for normalization of the results. The relative gene expression levels between mutant strain and the parent strain were analyzed using the qualified Ct (2^−ΔΔCt^) [40]. Primers used in this experiment are listed Additional file 1: Table S2.

### Statistical analysis

All experiments were conducted in triplicate and data were expressed in mean values and standard deviations. Results were analyzed via a software of GraphPad Prism 8, and p < 0.05 was regarded as statistically significant.

## Supplementary Information

**Additional file 1.** Additional file figures. **Figure S1. **HPLC analysis of extracellular oxalic acid and citric acid in *A. niger* S1075; **Figure S2.** Screening of transformants of *A. niger* S1075 with *cadA* overexpression; **Figure S3. **Screening of transformants of *A. niger* S1361 with *mttA *overexpression; **Figure S4.** Screening of transformants of *A. niger* S1486 with *mfsA *overexpression; **Figure S5.** Screening of transformants of *A. niger* S1075 with *adi1* and *tad1 *co-overexpression; **Figure S6.** Screening of transformants of *A. niger* S1683 with *mtt1 *overexpression; **Figure S7.** Screening of transformants of *A. niger* S1738 with *itp1 *overexpression; **Figure S8.** Screening of transformants of *A. niger* S1596 with *adi1 *and *tad1 *co-overexpression; **Figure S9.** Screening of transformants of *A. niger* S1779 with *itp1* overexpression; **Figure S10.** Screening of transformants of *A. niger* S2083 with increased *cadA *gene copy number; **Figure S11.** Screening of transformants of *A. niger* S2288 with *acoA *overexpression. Additional file tables. **Table S1. **Extracellular organic acid (mM) in different strains at 4-day and 6-day fermentation;** Table S2.** Primers used in this study.

## Data Availability

The datasets used and analyzed during the current study are available from the corresponding author upon reasonable request.

## Abbreviations

*cadA*: *Cis*-Aconitate decarboxylase gene
*mttA*: Mitochondrial transporter gene
*mfsA*: A putative major facilitator superfamily transporter gene
*adi1*: Aconitate-Δ-isomerase gene
*tad1*: *Trans*-Aconitate decarboxylase gene
*mtt1*: Mitochondrial transporter gene
*itp1*: Itaconate transporter protein gene
*cexA*: Citrate exporter gene
*oahA*: Oxaloacetate acetylhydrolase gene
*acoA*: Aconitase gene
*ictA*: Itaconyl-CoA transferase gene
P*gpd*A: Glyceraldehyde-3-phosphate dehydrogenase promoter
OE: Overexpression
IA: Itaconic acid
Ct: The calculated threshold cycle
PDA: Potato dextrose agar
LB: Luria Bertani
PEG: The polyethylene glycol
MES: Morpholinoethanesulfonic acid
DOX: Doxycycline
HPLC: High-performance liquid chromatography
