# Supplementary material for: Synergistic effects on itaconic acid production in engineered Aspergillus niger expressing the two distinct biosynthesis clusters from Aspergillus terreus and Ustilago maydis
Source: Microb Cell Fact. 2022 Aug 11;21:158. doi: 10.1186/s12934-022-01881-7 (PMC9367143; doi:10.1186/s12934-022-01881-7)
Supplement: Supplementary file 1 — Additional file 1. Additional file figures. Figure S1. HPLC analysis of extracellular oxalic acid and citric acid in A. niger S1075; Figure S2. Screening of transformants of A. niger S1075 with cadA overexpression; Figure S3. Screening of transformants of A. niger S1361 with mttA overexpression; Figure S4. Screening of transformants of A. niger S1486 with mfsA overexpression; Figure S5. Screening of transformants of A. niger S1075 with adi1 and tad1 co-overexpression; Figure S6. Screening of transformants of A. niger S1683 with mtt1 overexpression; Figure S7. Screening of transformants of A. niger S1738 with itp1 overexpression; Figure S8. Screening of transformants of A. niger S1596 with adi1 and tad1 co-overexpression; Figure S9. Screening of transformants of A. niger S1779 with itp1 overexpression; Figure S10. Screening of transformants of A. niger S2083 with increased cadA gene copy number; Figure S11. Screening of transformants of A. niger S2288 with acoA overexpression. Additional file tables. Table S1. Extracellular organic acid (mM) in different strains at 4-day and 6-day fermentation; Table S2. Primers used in this study. [file 12934_2022_1881_MOESM1_ESM.doc]

**Title:**

Synergistic effects on itaconic acid production in engineered *Aspergillus niger* expressing the two distinct biosynthesis clusters from *Aspergillus terreus* and *Ustilago maydis*

**Journal Name:**

Microbial Cell Factories

**Authors:**

Yaqi Wang^1^, Yufei Guo^1^, Wei Cao^1,2,3^, Hao Liu^1,2,3*^

**Affiliations:**

^1^MOE Key Laboratory of Industrial Fermentation Microbiology, College of Biotechnology, Tianjin University of Science & Technology, Tianjin 300457, China.

^2^Tianjin Engineering Research Center of Microbial Metabolism and Fermentation Process Control, Tianjin University of Science & Technology, Tianjin 300457, China.

^3^ National Technology Innovation Center of Synthetic Biology, 300308, Tianjin, P. R. China.

*** Corresponding Author:**

Phone: (86)-22-60600810. Fax: (86)-22-60602298. Email: liuhao@tust.edu.cn

**Table S1** Intracellular organic acid (mg/mg) in different strains at 6-day fermentation

| **Strains** | ***cis*-aconitate** | ***trans*-aconitate** |
| --- | --- | --- |
| S1075 | (0.72±0.08)×10^-4^ | Not detected |
| S1596 | (0.82±0.02)×10^-4^ | (0.10±0.04)×10^-4^ |
| S2120 | (0.78±0.04)×10^-4^ | (0.16±0.02)×10^-4^ |

**Table S2** Primers used in this study

| **Primer name** | **Name** | **Sequence （5’→3’）** |
| --- | --- | --- |
| Primers for verification of *hph* gene | | |
| Primer 641 | *hph*-upR | CAATATCAGTTAACGTCGAC |
| Primer 642 | *hph*-downF | GGAACCAGTTAACGTCGAAT |
| Primers for qRT-PCR of actin | | |
| Primer 992 | *actin*F | TCCTCACCCTCAGATACCC |
| Primer 993 | *actin*R | CACCGTCACCAGAGTCCA |
| Primers for amplification of *acoA* | | |
| Primer 2105 | *acoA*F | *ACTTCTCATCCATCTTCAAA*GAATTCATGGCCACCGTCACCGAC |
| Primer 2106 | *acoA*R | *GTGGATCCCTGCAGGGTACC*GAGCTCGCTTGATGTCGAAGGAGGC |
| Primers for amplification of *adi1* expression cassette | | |
| Primer 3210 | P*gpd*AF | *GCTATACGAAGTTAT*AAGCTTGAGACTAGTGGACTAACATTAT |
| Primer 3211 | T*trpC*F | *ACGACGGCCAGTGCC*AAGCTTGTCTAGAAAGAAGGATTACCTC |
| Primers for amplification of *cadA* | | |
| Primer 3501 | *cadA*F | *ATGGAATTCGAGCTC*GGTACCATGACCAAACAATCTGCGGAC |
| Primer 3502 | *cadA*R | *AGTGGATCCCTGCAG*GGTACCTTATACCAGTGGCGATTTCACG |
| Primers for amplification of *ictA-*5’f | | |
| Primer 3522 | *ictA*-upF | *CCCAGAATTCAATTC*GAGCTCGATGATCGTCAAGCGAGTTC |
| Primer 3523 | *ictA*-upR | *ATTATACGAAGTTAT*GGATCCGCAAAGTGTGTTGTTCCCA |
| Primers for amplification of *ictA-*3’f | | |
| Primer 3524 | *ictA*-downF | *CGAAGTTATTCTAGA*ACTAGTAATCCAATTCAGGCAACCTAC |
| Primer 3525 | *ictA*-downR | *TGCCTGCAGGGGCCC*ACTAGTGTTTCGCATGTCTCTGGC |
| Primers for qRT-PCR of *cadA* | | |
| Primer 4455 | *cadA*F mRNA | ACGGTATTGCATGTGCCTGG |
| Primer 4456 | *cadA*R mRNA | GCAGAGTGTAGGGGGGCTT |
| Primers for qRT-PCR of *mttA* | | |
| Primer 4457 | *mttA*F mRNA | CCCCTTTGAGTCTGCCAAGAC |
| Primer 4458 | *mttA*R mRNA | CACCAGTGGACAGCTCACCA |
| Primers for qRT-PCR of *mfsA* | | |
| Primer 4459 | *mfsA*F mRNA | CGACTTATGCTGTGGCCTCC |
| Primer 4460 | *mfsA*R mRNA | CTGAGGGAGACGGAATCCGC |
| Primers for qRT-PCR of *adi1* | | |
| Primer 4461 | *adi1*F mRNA | GCTGCACCCCATTGACACC |
| Primer 4462 | *adi1*R mRNA | CTCGGAGCGCTGAGTGGA |
| Primers for qRT-PCR of *tad1* | | |
| Primer 4463 | *tad1*F mRNA | CTCAAGCTCACCGGCTTGC |
| Primer 4464 | *tad1*R mRNA | CGATCTCTTGGACGCGGC |
| Primers for qRT-PCR of *mtt1* | | |
| Primer 4465 | *mtt1*F mRNA | GTCCGCTTCTTCGCCTTCG |
| Primer 4466 | *mtt1*R mRNA | GGACAATCGAGGCGGTGC |
| Primers for qRT-PCR of *itp1* | | |
| Primer 4467 | *itp1*F mRNA | CCGATGACGCTGCTCTGG |
| Primer 4468 | *Itp1*R mRNA | CTCGGAGGTGTGCAGCATG |

Underlined nucleotides represent the restriction enzyme recognition sites. Italic sequences indicate the cloned homologous sequences.

**
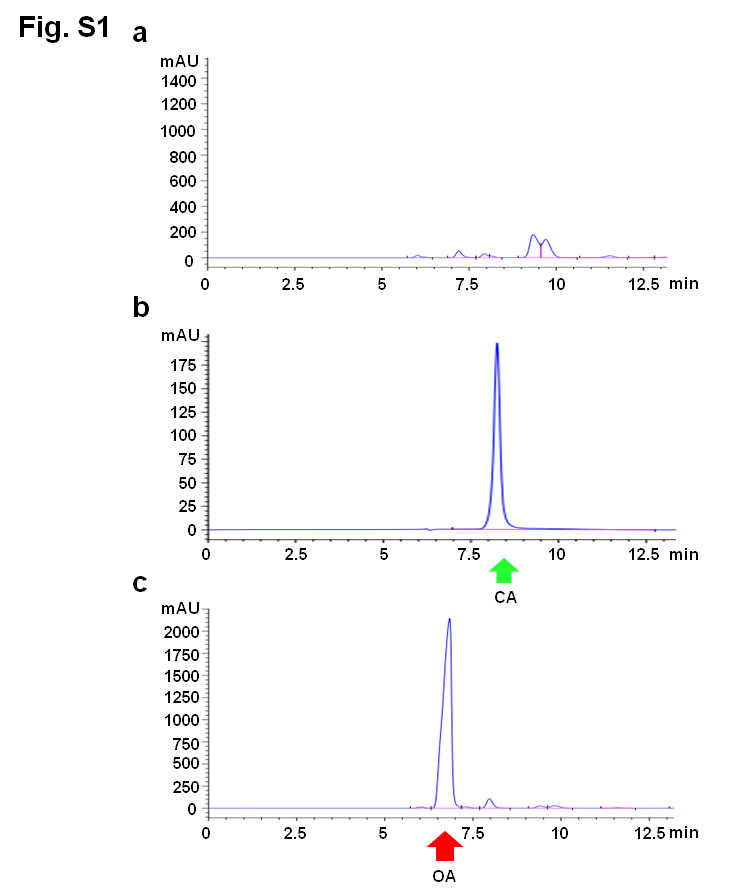
**

**
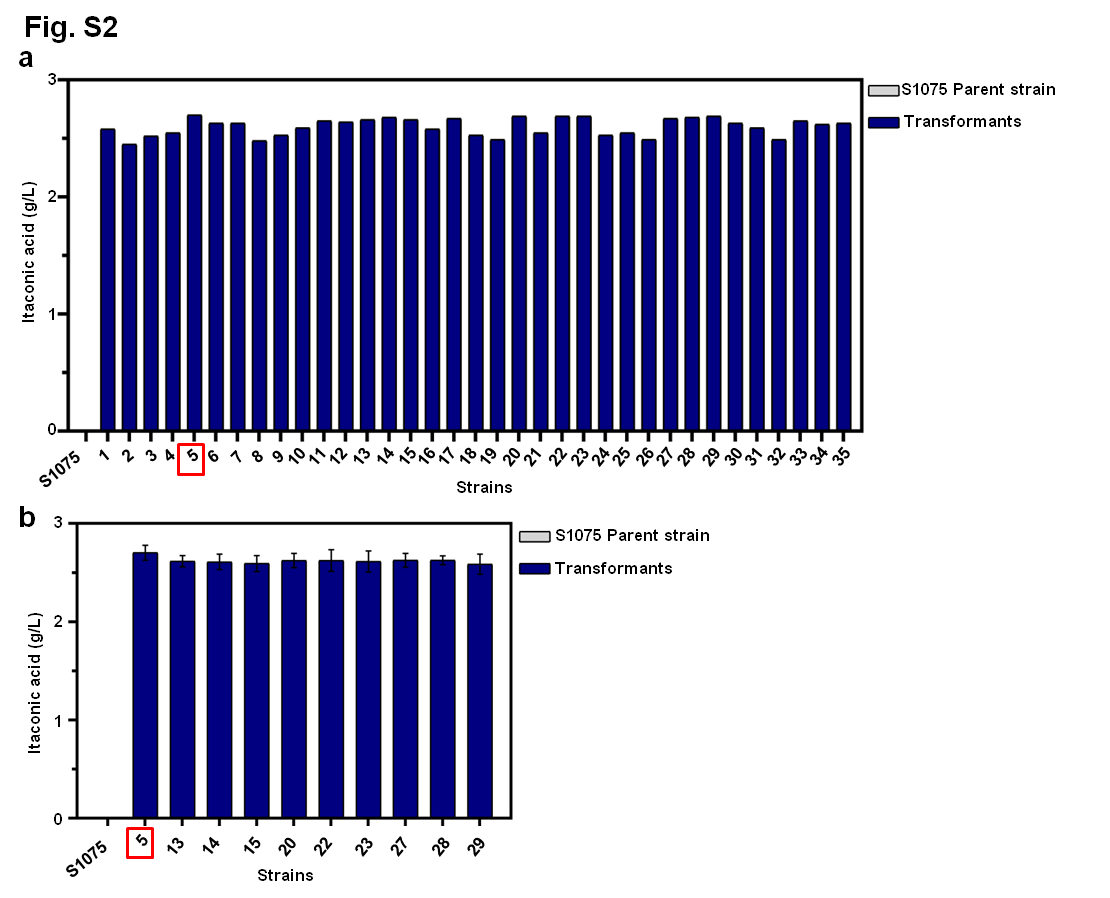
**

**
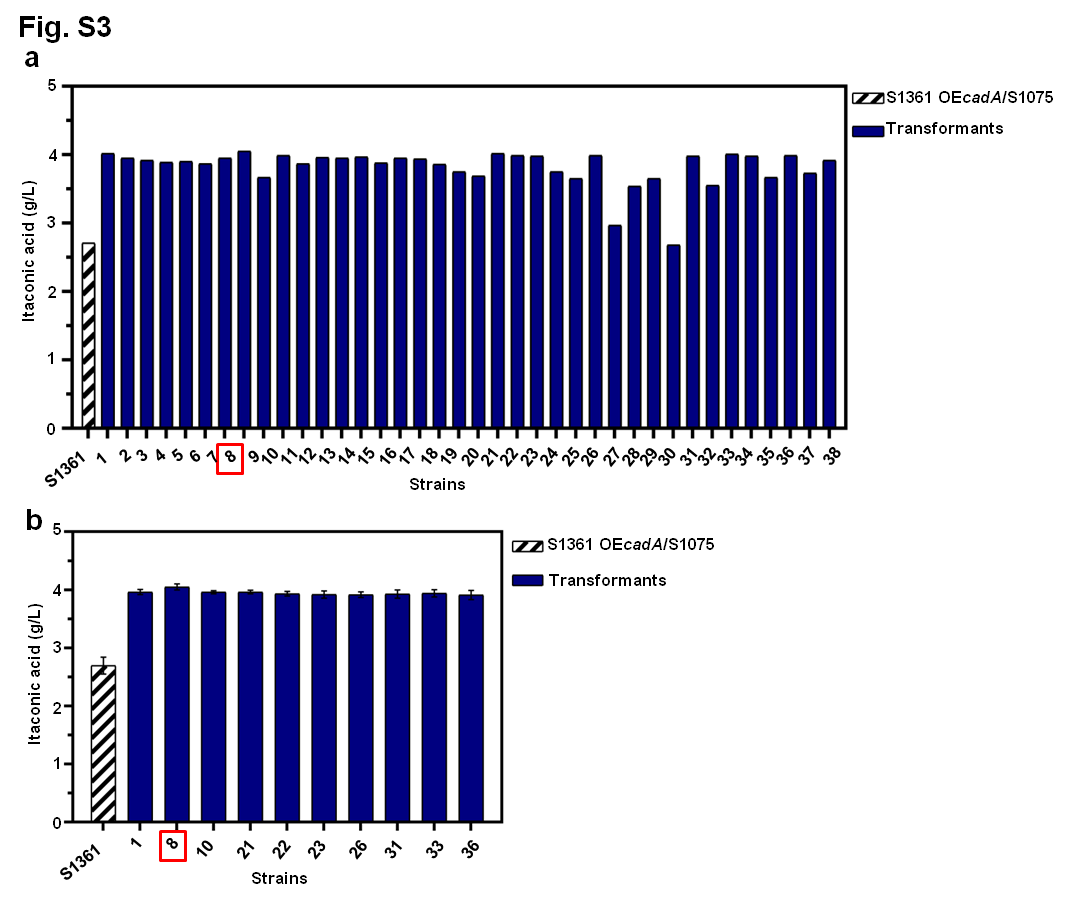
**

**
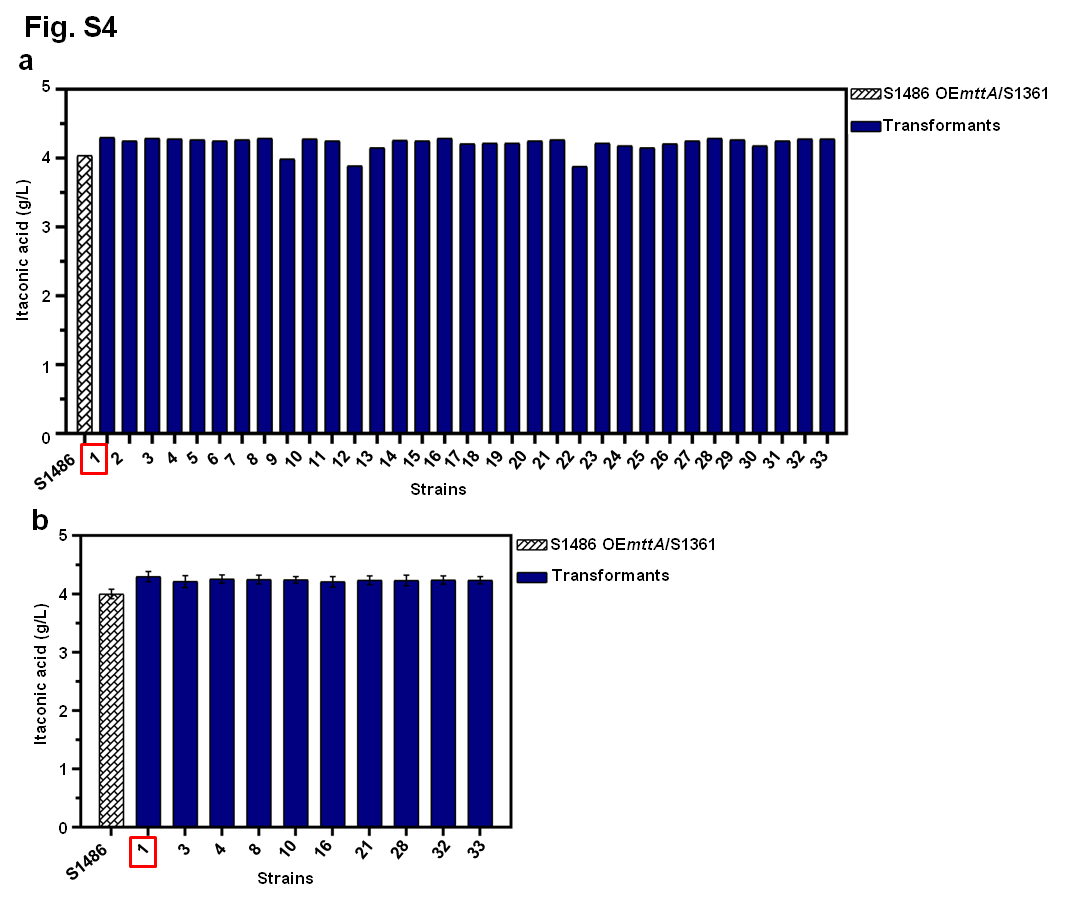
**

**
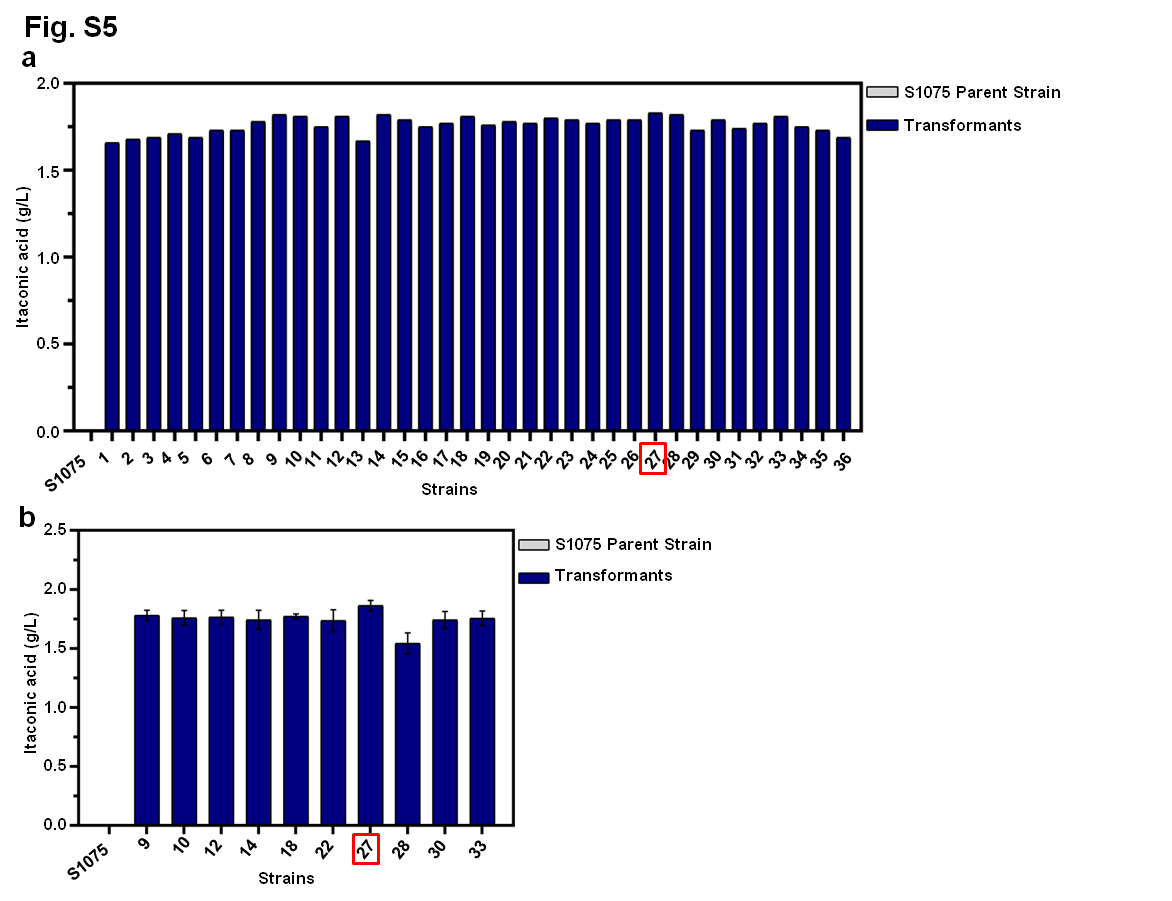
**

**
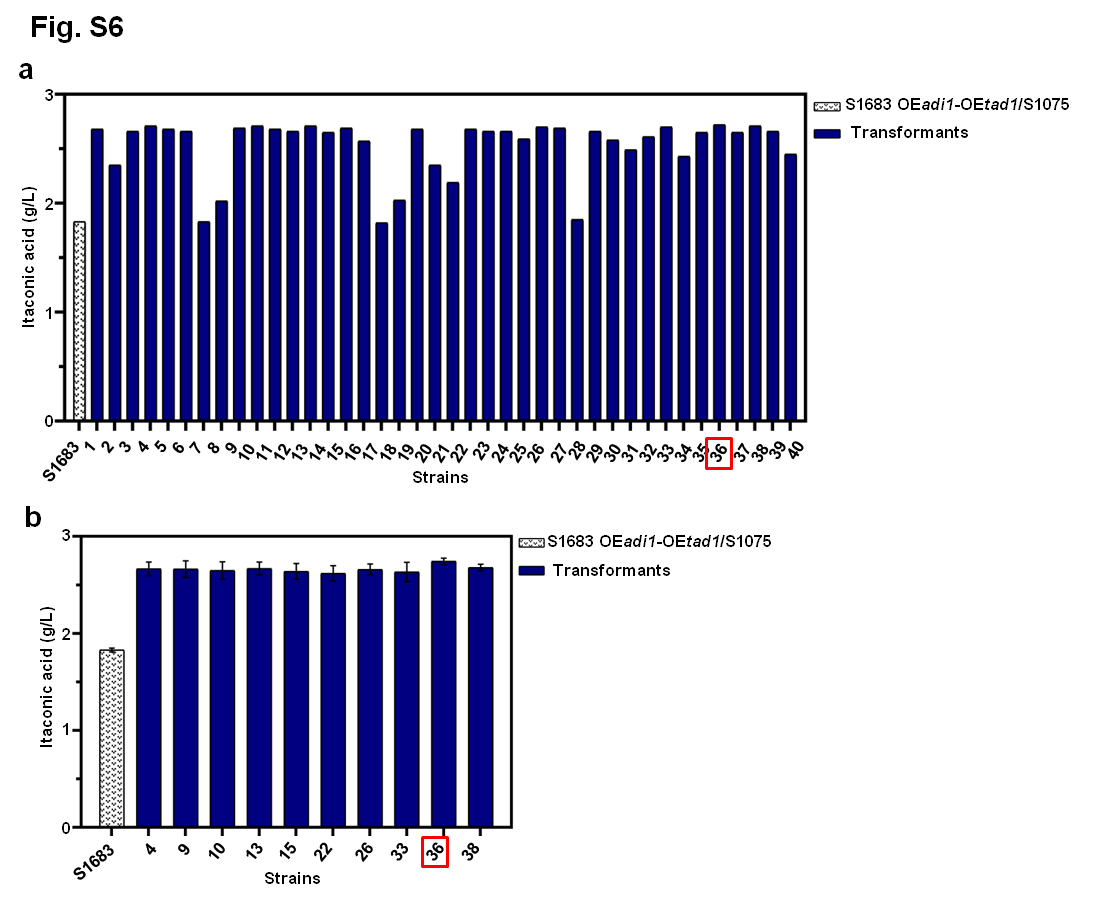
**

**
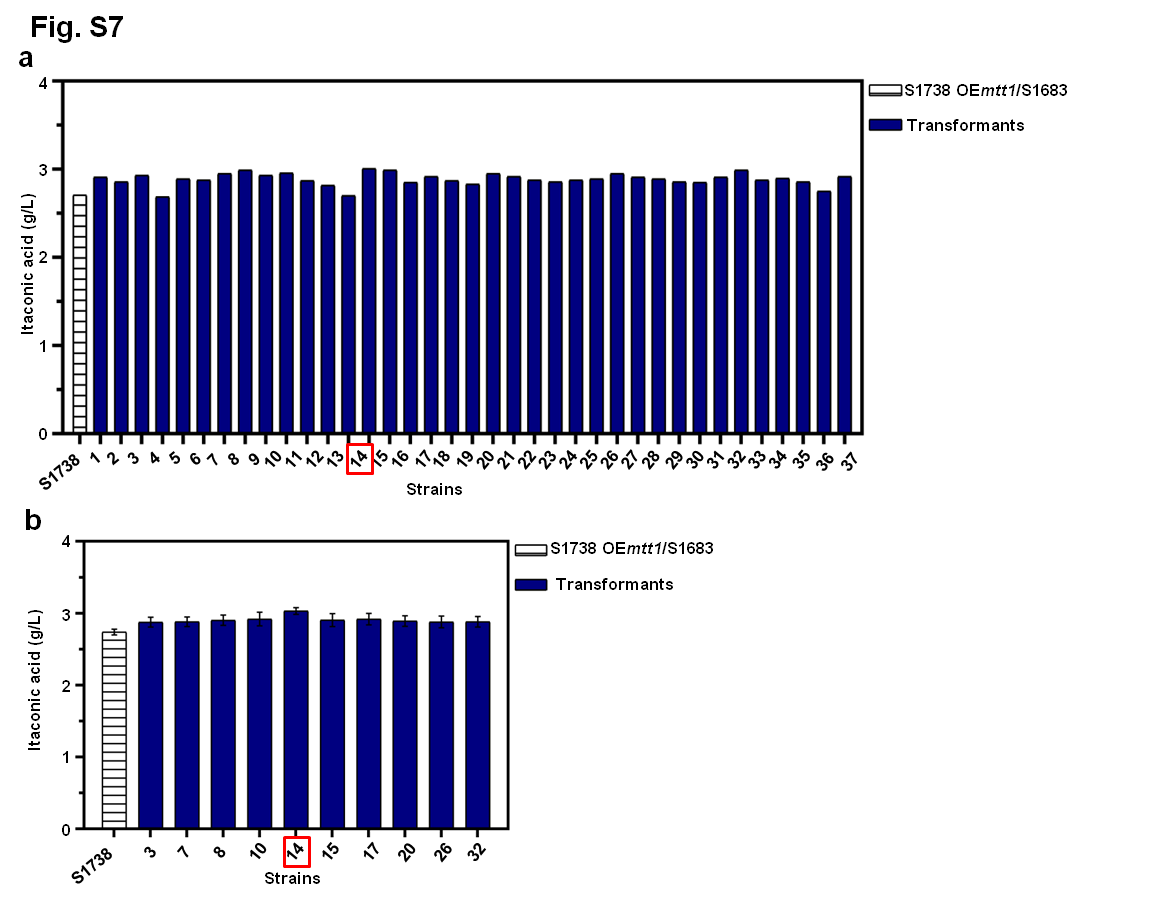
**

**
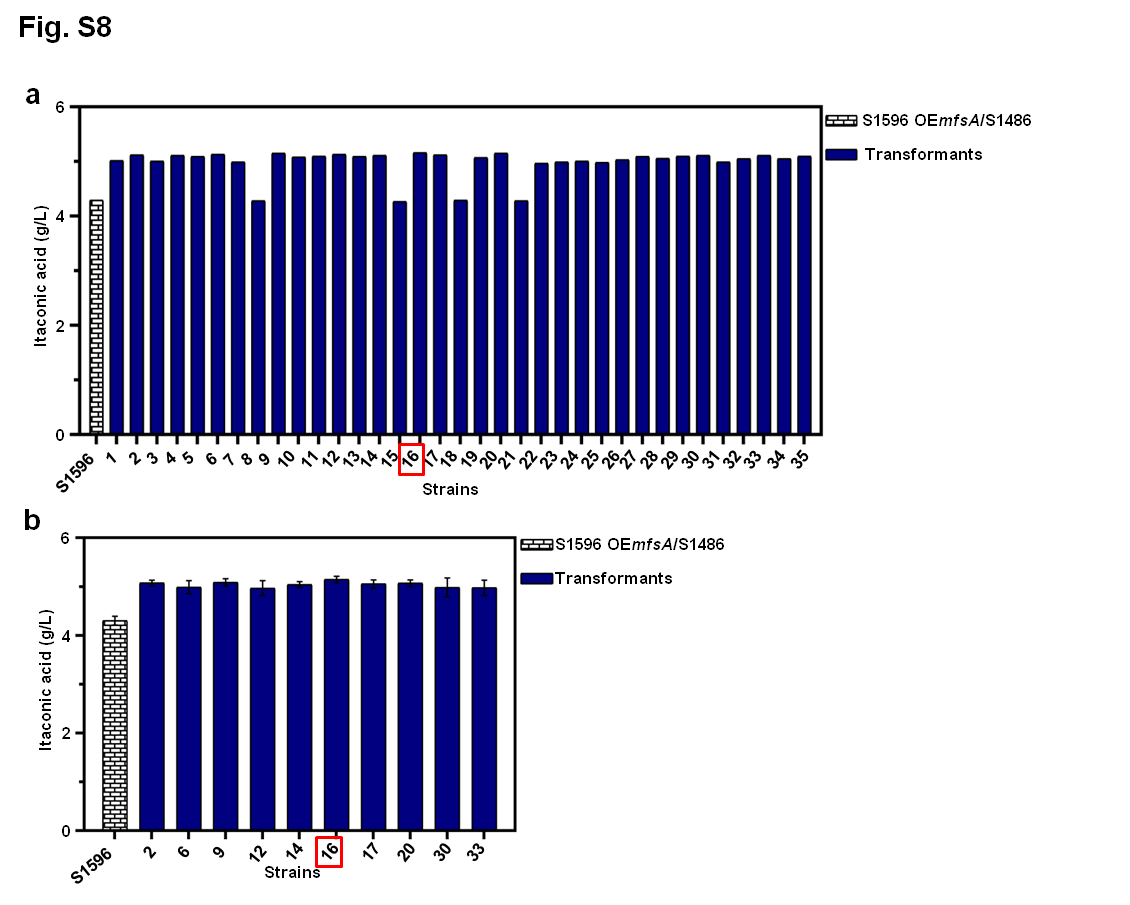
**

**
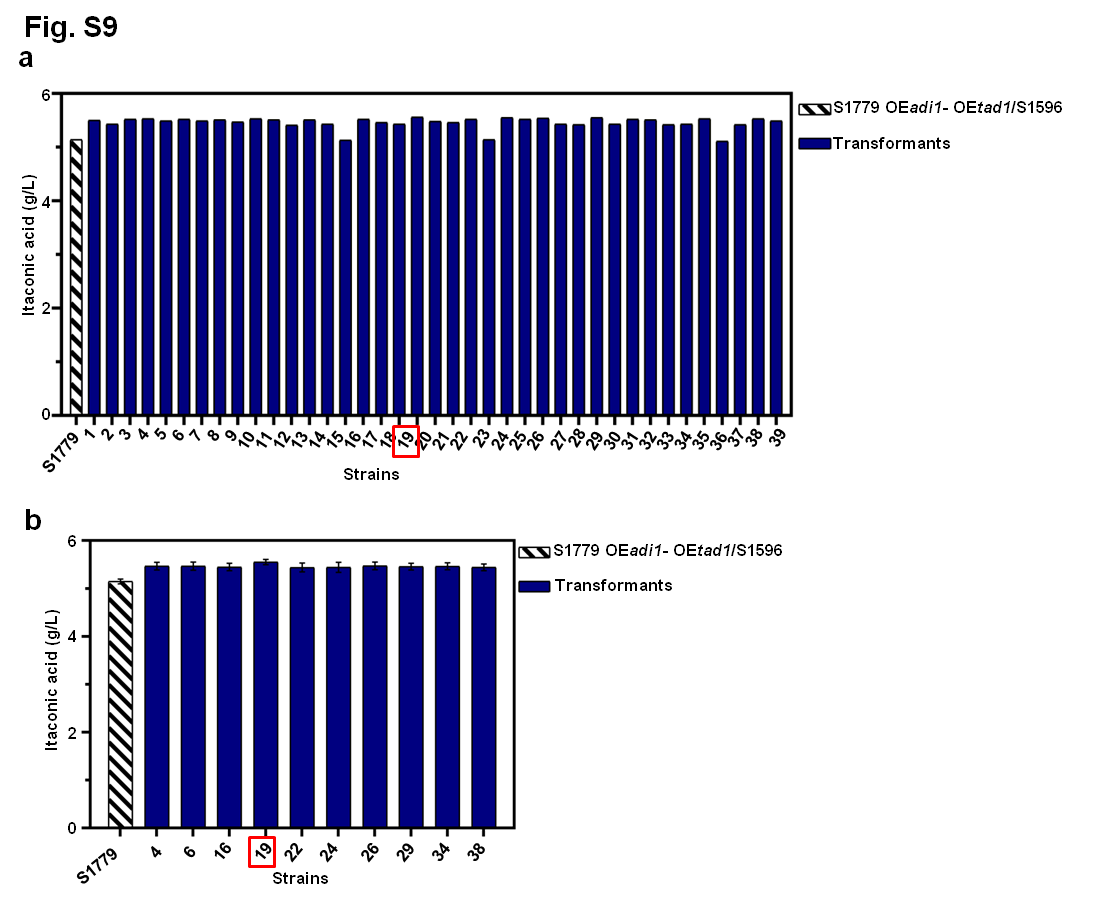
**

**
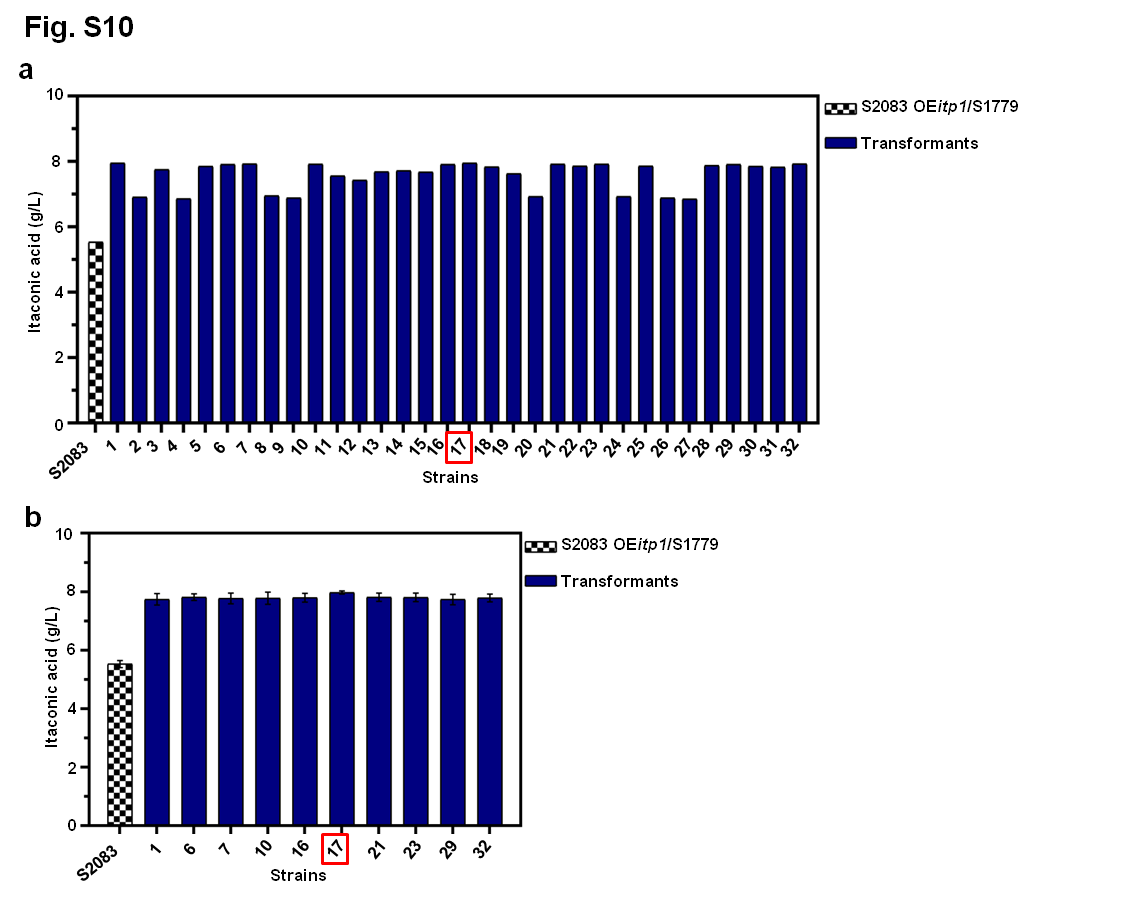
**

**
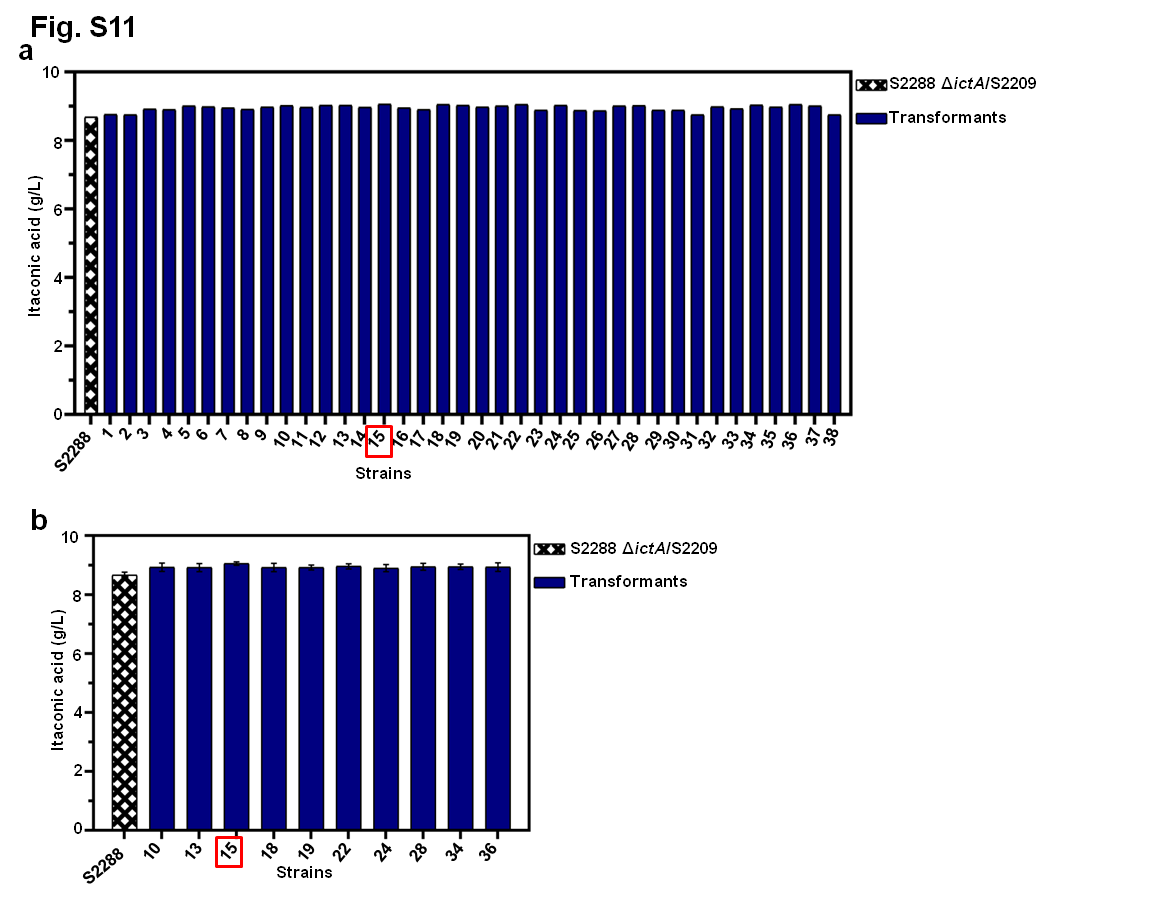
**

**Figure Legends:**

**Fig. S1** HPLC analysis of extracellular oxalic acid and citric acid in *A. niger* S1075. **a** *A. niger* S1075. **b** Citric acid standard. **c** Cxalic acid standard. Red arrow: oxalic acid (6.8 min). Green arrow: citric acid (7.9 min).

**Fig. S2** Screening of transformants of *A. niger* S1075 with *cadA* overexpression. **a** Primary screening of *cadA* overexpressing transformants of *A. niger* S1075 at 6-day in shake flask. 1~35: different transformants. **b** The second round of screening of *cadA* overexpressing transformants of *A. niger* S1075 at 6-day in shake flask. Red frame: the transformant with the highest IA production.

**Fig. S3** Screening of transformants of *A. niger* S1361 with *mttA* overexpression. **a** Primary screening of *mttA* overexpressing transformants of *A. niger* S1361 at 6-day in shake flask. 1~38: different transformants. **b** The second round of screening of *mttA* overexpressing transformants of *A. niger* S1361 at 6-day in shake flask. Red frame: the transformant with the highest IA production.

**Fig. S4** Screening of transformants of *A. niger* S1486 with *mfsA* overexpression. **a** Primary screening of *mfsA* overexpressing transformants of *A. niger* S1486 at 6-day in shake flask. 1~33: different transformants. **b** The second round of screening of *mfsA* overexpressing transformants of *A. niger* S1486 at 6-day in shake flask. Red frame: the transformant with the highest IA production.

**Fig. S5** Screening of transformants of *A. niger* S1075 with *adi1* and *tad1* co-overexpression. **a** Primary screening of *adi1* and *tad1* co-overexpressing transformants of *A. niger* S1075 at 6-day in shake flask. 1~36: different transformants. **b** The second round of screening of *adi1* and *tad1* co-overexpressing transformants of *A. niger* S1075 at 6-day in shake flask. Red frame: the transformant with the highest IA production.

**Fig. S6** Screening of transformants of *A. niger* S1683 with *mtt1* overexpression. **a** Primary screening of *mtt1* overexpressing transformants of *A. niger* S1683 at 6-day in shake flask. 1~40: different transformants. **b** The second round of screening of *mtt1* overexpressing transformants of *A. niger* S1683 at 6-day in shake flask. Red frame: the transformant with the highest IA production.

**Fig. S7** Screening of transformants of *A. niger* S1738 with *itp1* overexpression. **a** Primary screening of *itp1* overexpressing transformants of *A. niger* S1738 at 6-day in shake flask. 1~37: different transformants. **b** The second round of screening of *itp1* overexpressing transformants of *A. niger* S1738 at 6-day in shake flask. Red frame: the transformant with the highest IA production.

**Fig. S8** Screening of transformants of *A. niger* S1596 with *adi1* and *tad1* co-overexpression. **a** Primary screening of *adi1* and *tad1* co-overexpressing transformants of *A. niger* S1596 at 6-day in shake flask. 1~35: different transformants. **b** The second round of screening of *adi1* and *tad1* co-overexpressing transformants of *A. niger* S1596 at 6-day in shake flask. Red frame: the transformant with the highest IA production.

**Fig. S9** Screening of transformants of *A. niger* S1779 with *itp1* overexpression. **a** Primary screening of *itp1* overexpressing transformants of *A. niger* S1779 at 6-day in shake flask. 1~39: different transformants. **b** The second round of screening of *itp1* overexpressing transformants of *A. niger* S1779 at 6-day in shake flask. Red frame: the transformant with the highest IA production.

**Fig. S10** Screening of transformants of *A. niger* S2083 with increased *cadA* gene copy number. **a** Primary screening of transformants of *A. niger* S2083 with increased *cadA* gene copy number at 6-day in shake flask. 1~32: different transformants. **b** The second round of screening of transformants of *A. niger* S2083 with increased *cadA* gene copy number at 6-day in shake flask. Red frame: the transformant with the highest IA production.

**Fig. S11** Screening of transformants of *A. niger* S2288 with *acoA* overexpression. **a** Primary screening of *acoA* overexpressing transformants of *A. niger* S2288 at 6-day in shake flask. 1~38: different transformants. **b** The second round of screening of *acoA* overexpressing transformants of *A. niger* S2288 at 6-day in shake flask. Red frame: the transformant with the highest IA production.
